# Editorial: Highly resolved spatio-temporal dynamics of genome organization and its link with transcriptional dynamics editorial

**DOI:** 10.3389/fmolb.2023.1213345

**Published:** 2023-06-22

**Authors:** Rajarshi P. Ghosh

**Affiliations:** ^1^ Howard Hughes Medical Institute, Chevy Chase, MD, United States; ^2^ Molecular and Cell Biology, University of California, Berkeley, Berkeley, CA, United States

**Keywords:** genomics, nucleosome, chromatin conformation, genome organization, genome dynamics

The genomic organization within cellular space constitutes a fundamental aspect of cellular information processing, as it governs genome dynamics and function. However, the challenge of relating local structural and biochemical features of chromatin to the global organization of the genome and its function has posed a formidable hurdle. This difficulty stems from the complex underlying biology and the lack of suitable tools that can track multiple components in complex cellular reactions over different time and length scales. Innovative tools are required to provide exquisite details about the structural states of nucleosomes at defined sites in the genome, generate dynamic connectivity maps of the genome at high resolution, allow researchers to eavesdrop on the process of chromatin remodeling in a kinetically well-defined manner, and reduce the cost of deep sequencing. With the ongoing advances in technology, such innovative tools have the potential to transform the field of chromatin research and facilitate the development of new therapies for a variety of human diseases.

The fundamental packing units of chromosomes are nucleosomes which are composed of ∼146 bp DNA tightly packed around an octamer of core histone proteins. The structure and dynamics of nucleosomes have been extensively studied, but little is known about the intermediate states of nucleosomes, especially those with partially disassembled histones. In an article titled “Histone tail dynamics in partially disassembled nucleosomes during chromatin remodeling” published as a part of this Research Topic, Kameda et al. using all-atom molecular dynamics simulations have shown that the intrinsic dynamics of DNA is markedly affected in nucleosomes lacking specific complements of histone dimers. This work underscores the need for developing new approaches to detect and analyze transient structural states of nucleosomes in the genome.

In recent years the auxin-inducible degron (AID) system has become indispensable in studying the role of chromatin architectural proteins in molding the genomic landscape. However, the efficacy of controlling protein concentration using the auxin-inducible degron (AID) system varies widely from protein to protein and in different organisms and cell types. Yunusova et al. conducted a study as a part of this Research Topic, titled “Evaluation of the OsTIR1 and AtAFB2 AID Systems for genome architectural protein degradation in mammalian cells.” The researchers compared the degradation dynamics of cohesin/condensin complex subunits in mouse embryonic stem cells and human haploid HAP1 line using two AFBs, OsTIR1 and AtAFB2. The study found that AtAFB2 was the more efficient of the two approaches. This comparative analysis will be greatly useful to researchers trying to select the appropriate AID system for their experimental model.

The field of genomics has witnessed remarkable progress in recent years, driven by the rapid increase in genome-sequencing throughput, improvements in library preparation methods, and the integration of single-molecule techniques. As a result, genomics tools have emerged as a crucial component of both clinical and fundamental research. Despite their numerous benefits, the routine use of these tools in everyday genomics operations remains limited by cost considerations. It is therefore important to strike a balance between the benefits of these advanced techniques and their associated costs in order to fully realize their potential. In a case study published as a part of this Research Topic titled “Benchmarking of ATAC Sequencing Data from BGI’s low-cost DNBSEQ-G400 instrument for identification of open and occupied chromatin regions” Naval-Sanchez et al. has used Assay for Transposase-Accessible Chromatin using sequencing (ATAC-seq) to benchmark data from a low-cost sequencer against data from a standard Illumina instrument, using the same bulk ATAC-seq libraries generated from pluripotent stem cells and fibroblasts. The study found that both platforms enabled comparable levels of open chromatin identification thus achieving a sequencing depth comparable to an Illumina platform but at a lower cost.

The field of genomics has undergone a rapid transformation in recent years with the advent of single-cell sequencing and genome-wide chromatin conformation technologies. These methods have enabled researchers to study the organization and folding of the genome at unprecedented resolution, allowing for a deeper understanding of how genes are regulated and expressed. In a review published as a part of this Research Topic titled “Every gene everywhere all at once: High-precision measurement of 3D chromosome architecture with single-cell Hi-C” Chi et al. discuss the impact of the advent of single-cell HiC methods in elucidating general principles of chromosome folding in single cell nucleus.

In this article, the authors provide an overview of the role of three-dimensional (3D) chromosome structure in regulating fundamental biological processes and how changes in the spatial genome organization may result in a spectrum of debilitating disorders. The authors discuss the limitations of traditional microscopy and biochemistry techniques in studying chromosome folding and introduce new technologies that have revolutionized the field of 3D genomics. The article highlights two main categories of single-cell 3D genomics technologies: imaging-based methods and sequencing-based methods. The focus of the article is on sequencing-based methods, particularly single-cell Hi-C (scHi-C), which in recent years has emerged as a powerful tool for mapping 3D contacts in individual genomes.

The authors discuss the improvements and advancements in scHi-C technology over the years, including increased sensitivity and scalability. They also highlight the discovery of chromatin domains, also known as topologically associating domains (TADs), which are contact-rich regions within chromosomes, and their variability at the single-cell level. The article further explores the concept of chromatin compartments, which are statistically defined regions of the genome based on contact profiles.

The authors emphasize the importance of single-cell approaches in uncovering the cell-to-cell variability in chromosome structure and its relationship to gene expression and genome function. They discuss the potential origins and functions of chromatin domains and compartments, as well as the challenges in studying and annotating them at the single-cell level.

Overall, the article provides a comprehensive overview of single-cell Hi-C and its applications in studying chromosome architecture, highlighting its ability to reveal features of 3D genome organization that are obscured by population averaging in ensemble Hi-C approaches and its impact on our understanding of DNA organization and function.

In addition to single-cell sequencing technologies, recent advancements in the fields of optogenetics, genome editing, polymer physics, and single-molecule imaging have contributed to the development of cutting-edge tools that enable researchers to explore cellular information processing with unprecedented precision. These advanced tools include novel molecular tags that allow for prolonged single-molecule tracking within cells, optogenetics-based methods to study nuclear condensates, multiplexed FISH platforms for spatial transcriptomics and genomics, kinetic analysis of transcription at the single-molecule level, and new microscopy capabilities that bridge different imaging modalities. These developments have the potential to revolutionize the way we investigate genomic organization and its impact on cellular function. By providing a deeper understanding of the intricate relationship between chromatin structure and cellular function, these advancements will help to unravel the complexities of gene regulation and pave the way for the development of new therapeutic strategies.

